# Molecular markers for analyses of intraspecific genetic diversity in the Asian Tiger mosquito, *Aedes albopictus*

**DOI:** 10.1186/s13071-015-0794-5

**Published:** 2015-03-28

**Authors:** Mosè Manni, Ludvik M Gomulski, Nidchaya Aketarawong, Gabriella Tait, Francesca Scolari, Pradya Somboon, Carmela R Guglielmino, Anna R Malacrida, Giuliano Gasperi

**Affiliations:** Department of Biology and Biotechnology “L. Spallanzani”, University of Pavia, Pavia, Italy; Department of Biotechnology, Mahidol University, Bangkok, Thailand; Department of Parasitology, Chiang Mai University, Chiang Mai, Thailand

**Keywords:** *Aedes albopictus*, Microsatellites, Simple sequence repeats (SSRs), Internal transcribed spacer 2 (ITS2), Mosquito, Virus vectors, Population genetics

## Abstract

**Background:**

The dramatic worldwide expansion of *Aedes albopictus* (the Asian tiger mosquito) and its vector competence for numerous arboviruses represent a growing threat to public health security. Molecular markers are crucially needed for tracking the rapid spread of this mosquito and to obtain a deeper knowledge of population structure. This is a fundamental requirement for the development of strict monitoring protocols and for the improvement of sustainable control measures.

**Methods:**

Wild population samples from putative source areas and from newly colonised regions were analysed for variability at the ribosomal DNA internal transcribed spacer 2 (ITS2). Moreover, a new set of 23 microsatellite markers (SSR) was developed. Sixteen of these SSRs were tested in an ancestral (Thailand) and two adventive Italian populations.

**Results:**

Seventy-six ITS2 sequences representing 52 unique haplotypes were identified, and AMOVA indicated that most of their variation occurred within individuals (74.36%), while only about 8% was detected among populations. Spatial analyses of molecular variance revealed that haplotype genetic similarity was not related to the geographic proximity of populations and the haplotype phylogeny clearly indicated that highly related sequences were distributed across populations from different geographical regions. The SSR markers displayed a high level of polymorphism both in the ancestral and in adventive populations, and *F*_ST_ estimates suggested the absence of great differentiation. The ancestral nature of the Thai population was corroborated by its higher level of variability.

**Conclusions:**

The two types of genetic markers here implemented revealed the distribution of genetic diversity within and between populations and provide clues on the dispersion dynamics of this species. It appears that the diffusion of this mosquito does not conform to a progressive expansion from the native Asian source area, but to a relatively recent and chaotic propagule distribution mediated by human activities. Under this scenario, multiple introductions and admixture events probably play an important role in maintaining the genetic diversity and in avoiding bottleneck effects. The polymorphic SSR markers here implemented will provide an important tool for reconstructing the routes of invasion followed by this mosquito.

## Background

The Asian tiger mosquito, *Aedes* (Stegomyia) *albopictus* is a vector of numerous arboviruses including Dengue, Chikungunya, and a variety of epizootic viruses [1-3]. It is considered to be the most invasive mosquito species in the world [4], and it is listed among the top hundred most dangerous invasive species (http://www.issg.org/database/species/search.asp?st=100ss).

Originally a native of the tropical forests of Southeast Asia, it has spread during the last 30–40 years to both tropical and temperate regions worldwide [4,5]. Global warming can result in wetter and warmer conditions that favour the spread of this mosquito and its aquatic larvae [6,7]. The dramatic expansion of this aggressive daytime biting mosquito has increased public health concerns for arbovirus-related disease outbreaks [8-11]. Some biological traits such as feeding preferences [12], winter diapause [13], and vector competence [14] of *Ae. albopictus* vary according to its geographic origin. Given that these traits may be genetically determined and may influence pathogen transmission, it is of paramount importance to define the geographic origin of invading populations.

Furthermore, as the threat represented by this mosquito is growing due to the lack of sustainable control measures and of the progressive spread of insecticide-resistance [15,16], efficient surveillance methods may depend on the detailed knowledge of the population structure and dynamics of this vector [10,17]. Moreover, the potential presence of cryptic subgroups, that may be unaffected by control methods, should not be under-estimated. Indeed, great variation in terms of genome size has been previously identified in strains from different regions [18-21]. Variations in the rDNA cistron have also been reported within and among populations of this mosquito [22]. In this context, the Internal Transcribed Spacer 2 (ITS2) of nuclear ribosomal DNA has been shown to be a useful marker, because of its power in detecting differentiation, not only among cryptic species [23-25], but also among conspecific populations [26-28]. As ITS2 is one of the less functionally restricted regions of the rDNA, it can accumulate mutations within isolated populations relatively quickly and can thus be an indicator of genetic discontinuity between populations [29,30]. The genetic diversity and the phylogeographic relationships among populations have been extensively analysed using mitochondrial DNA (mtDNA) markers [31-35]. The data from populations collected in various regions of the world were consistent in displaying the presence of weak sequence variation at mtDNA loci, probably due to the recent spread of the species, but also due to the low mutation rate of these markers. Indeed, when a set of 13 microsatellite markers were used to analyse the invasion of the Australian region [36], they appeared to be more informative, compared to mtDNA markers, in resolving the population structure and the invasion dynamics. Moreover, considering that *Ae. albopictus* populations are infected by the symbiont *Wolbachia* [37], whose presence can impact mtDNA diversity within host populations, the utility of mtDNA as a neutral marker is reduced [38].

In the present study we used the ITS2 region to address the presence of intraspecific differentiation (genetic barriers) among groups of populations from different ancestral and recently colonized geographic areas. Moreover, we developed additional novel microsatellite markers for this mosquito species. We provide evidence of their usefulness for the analysis of the genetic composition of populations.

## Methods

### Mosquito samples

Nine wild population samples of *Ae. albopictus* were collected across the species range (Table 1). Two samples were from Thailand (Ban Rai, Lampang Province in the North and Phato, Chumphon Province in the South); two from Réunion (St. Denis in the North and St. Pierre in the South-West); and five from Northern Italy (Brescia, Castellanza, Pavia, Modena, Cesena). The Thai samples are from a region that pertains to the putative home range of the species, while Réunion and the Italian samples are representative of old (17th and 18th century) [39] and recently colonized (1990) [40] areas, respectively. For each of the samples, eggs were collected using ovitraps and the emerging adults were reared in the insectary at 27°C, with 60-80% relative humidity and a 12:12 h (L:D) photoperiod. The identities of all the samples were confirmed using the morphological keys of Rueda [41]. The nine population samples were screened for sequence variability of the ITS2 marker.

**Table 1:** ***Aedes albopictus*** **wild population samples**

**Region**	**Sample**	**Latitude**	**Longitude**	**Collection date**
Thailand	Ban Rai	18.31	99.55	November 2010
	Phato	9.84	98.79	October 2010
Réunion	St. Denis	−20.88	55.45	March 2010
	St. Pierre	−21.33	55.47	March 2010
Italy	Brescia	45.54	10.22	July 2010
	Pavia	45.19	9.16	October 2010
	Castellanza	45.61	8.89	October 2010
	Cesena	44.20	12.40	August 2010
	Modena	44.65	10.92	October 2009

The Rimini laboratory strain (F35) was used for the construction of the microsatellite-enriched libraries. This strain was established from mosquitoes collected in Rimini (Emilia Romagna) at the Centro Agricoltura Ambiente ‘G. Nicoli’ (Crevalcore, Italy). Three of the nine wild samples previously described, Ban Rai, Cesena and Brescia, were also screened to assess the variability of the newly developed microsatellites markers.

### ITS2 marker characterization

DNA was extracted according to Baruffi et al. [42] from individual females of the population samples. The extracted DNA was resuspended in TE (10 mM Tris–HCl, pH 8, 1 mM EDTA) and its concentration was quantified using a Nanodrop ND-1000 spectrophotometer (Nano-drop Technologies Inc., Wilmington, DE, USA). The ribosomal DNA ITS2 region was amplified from the DNA samples using the universal primers 5.8Sf 5′-atcactcggctcgtggatcg-3′ and 28Sr 5′-atgcttaaatttagggggtagtc-3′ [43]. These primers anneal to highly conserved sequences in the 5.8S and 28S rDNA genes flanking the ITS2 region. Reactions were performed in a volume of 25 μl with approximately 3 ng DNA, 1.5 mM MgCl_2_, Reaction buffer (10 mM Tris–HCl pH 9, 50 mM KCl), 200 µM dNTP, 10 pmol of each primer and 1 unit *Taq* DNA polymerase (Invitrogen). Amplifications were performed using an Eppendorf MasterCycler with the following cycle conditions: an initial denaturing step at 94°C for 2 min; twenty-five cycles of denaturation at 94°C for 20 s, annealing at 54°C for 15 s, extension at 72°C for 25 s, followed by a final extension at 72°C for 5 min.

The PCR products were separated on 1.5% agarose gels, stained with ethidium bromide and photographed under ultraviolet light. Amplification products were ligated into the TOPO 2.1 vector and transformed into competent One Shot cells using the TOPO TA cloning kit (Invitrogen). DNA inserts were sequenced on both strands.

### ITS2 data analysis

The boundaries of the ITS2 region were determined by comparison with the available *Ae. albopictus* sequences in GenBank. The ITS2 sequences were aligned using MUSCLE 3.8 [44]. Nucleotide diversity and gene diversity parameters were determined using Arlequin 3.5.1.2 [45]. Geographic structure in the ITS2 data set was investigated by spatial analyses of molecular variance implemented in SAMOVA 1.0 [46]. This approach identifies groups of populations that are genetically and geographically homogeneous and maximally differentiated from each other. The method requires the *a priori* definition of the number of groups (*K*) of populations that exist and generates *F*-statistics (*F*_SC_, *F*_ST_ and *F*_CT_) using an AMOVA approach. Different numbers of groups (*K*) were tested, and a simulated annealing procedure permitted the identification of the composition of each of the *K* groups that maximizes the *F*_CT_ index (proportion of total genetic variance due to differences between groups). The program was run for two to eight groups (*K* = 2 to *K* = 8) each time with the simulated annealing process repeated 100 times, starting each time with a different partition of the population samples into the *K* groups. A phylogenetic analysis was also performed including the unique ITS2 sequences or haplotypes identified in the nine population samples by Arlequin 3.5.1.2 [45]. The haplotype sequences together with an *Ae. flavopictus* ITS2 outgroup sequence (GenBank accession number: AF353559.1) were aligned using MUSCLE 3.8 [44]. The Kimura-2-parameter and Γ distribution model indicated by the Akaike Information Criterion (AIC = 3064) and the Bayesian Information Criterion (BIC = 3899), was used to perform Maximum Likelihood analyses in MEGA 5.2.2 [47]. The reliability of the resulting haplotype tree was determined by 1000 bootstrap replications. The mid-point rooted tree was drawn using FigTree v1.4 (http://tree.bio.ed.ac.uk/software/figtree/).

### Microsatellite isolation and characterization

Total genomic DNA was extracted individually from 64 mosquitoes (40 males and 24 females) from the Rimini strain using the method reported by Baruffi et al. [42]. The DNA was subsequently pooled and submitted to Genetic Identification Services, GIS (Chatsworth, CA, USA, http://www.genetic-id-services.com) for library construction. The library was enriched for four different di- and tri-nucleotide microsatellite motifs, namely (CA)n, (GA)n, (AAC)n, and (ATG)n. Insert DNA from individual clones was amplified by PCR following GIS guidelines. The PCR products were purified using QIAquick columns (Qiagen) and sequenced using the DYEnamic ET Terminator Cycle Sequencing Kit (Amersham Bioscience) with the universal M13 primers (M13f 5′-aggaaacagctatgaccatg-3′ and M13r 5′-acgacgttgtaaaacgacgg-3′). Clones with inserts less than 200 bp were not considered for sequencing. Sequencing products were resolved on the Applied Biosystems model 377 DNA Sequencer (Applied Biosystems). The resulting sequences were screened for the presence of microsatellite motifs using WebSat [48]. All the clone sequences were checked for redundancy using BLASTn [49].

Microsatellite sequences that shared one or both the 5′/3′ flanking regions in common were discarded to exclude potential multi-locus microsatellites families. The sequences that were found to be unique within the library were subjected to BLASTn analysis against the nr database to identify eventual hits with previously described sequences. Primer pairs were designed for the flanking regions of the identified SSR sequences using Primer 3 [50]. Preliminary PCR screenings were performed on 20 individuals (10 males and 10 females) from the Rimini strain to validate the primer efficiencies in single SSR locus amplifications. DNA amplifications were performed on a Mastercycler gradient (Eppendorf). The PCR reaction mixture consisted of 50 ng genomic DNA, 1x PCR buffer, 1.5 mM MgCl_2_, 270 µM dNTPs (Invitrogen), 1 U *Taq* polymerase (Invitrogen), and 10 μM of each primer, one of which was 5′ labelled with a fluorescent dye, in a final volume of 15 μl. PCR cycling conditions were 94°C for 3 min, 30 cycles of 94°C for 30 s, 57-60°C for 30 s, and 72°C for 30 s, followed by a final extension step of 72°C for 10 minutes. Aliquots of PCR products were separated by electrophoresis on 2% agarose gels stained with ethidium bromide, and visualized under UV light. Each PCR product was then diluted 1:10 in ddH_2_O water and 1 μl of this dilution was added to 9 μl of a mixture of deionized formamide and GeneScan-500 ROX size standard (Applied Biosystems). After denaturation for 4 min at 94°C the fragments were resolved on an ABI PRISM 310 Genetic Analyzer (Applied Biosystems) with the GENESCAN software package (Applied Biosystems). To avoid genotyping errors, the program TANDEM [51] was used to automate binning of the microsatellite allele length variants.

### Microsatellite data analysis

The parameters of intra- and inter-population genetic diversity, such as observed allele size, number of actual alleles (n_a_), number of effective alleles (n_e_), number of private alleles (n_p_) and AMOVA were computed using GenALEx 6.5 [52]. Observed and expected heterozygosity (H_O_ and H_E_, respectively), gene diversity (H_S_) and *F*_ST_ values were computed using MICROSATELLITE ANALYSER (MSA) V.4.05 [53]. Linkage disequilibrium, the frequency of null alleles (A_n_) and the deviations from Hardy-Weinberg expectations were computed using GENEPOP version 4.2 [54,55]. For loci with fewer than five alleles, an exact test of Hardy-Weinberg proportions was performed. For loci with five alleles or more, an unbiased estimate of the exact probability was obtained using the Markov chain method of Guo & Thompson [56] for each combination of locus and population. The allelic Polymorphic Information Content (PIC) was derived using CERVUS [57].

## Results

### ITS2 sequence variability

ITS2 sequences were obtained from an average of three individuals from each of the nine population samples collected in Thailand, Réunion and Italy (Table 2). In addition, to determine whether multiple copies of the ITS2 region varied within individuals, up to four clones were sequenced from each of these individuals. The primers amplified a product of 502–588 bp in length that included part of 5.8S and 28S sequences. The ITS2 sequence itself ranged from 322 to 408 bp in length with an average GC composition of 51%. The 76 ITS2 sequences, obtained from a total of 26 individuals, represent 52 unique sequence-types or haplotypes (Table 2). The gene diversity estimates were very high across the analysed sequences and ranged from 0.893 ± 0.111 in St. Denis (Réunion) and Castellanza (Italy) to 1.000 ± 0.052 in Brescia (Italy). Nucleotide diversity (π), was very low, ranging from 0.005 ± 0.004 in St. Pierre (Réunion) to 0.0191 ± 0.012 in Cesena (Italy). These low nucleotide diversity estimates are expected, as in the 415 bp MUSCLE ITS2 alignment only 44 sites (~10%) contained substitutions.

**Table 2:** **Features of the internal transcribed spacer 2 (ITS2) sequences in the different** ***Aedes albopictus*** **population samples**

**Region**	**Sample**	**Individuals analysed**	**Sequences analysed**	**Haplotypes present**	**Gene diversity ± SD**	**Nucleotide diversity (π ± SD)**
Thailand	Ban Rai	3	9	7	0.917 ± 0.092	0.0166 ± 0.0098
	Phato	4	13	9	0.936 ± 0.051	0.0128 ± 0.0075
Réunion	St. Denis	3	8	6	0.893 ± 0.111	0.0129 ± 0.0080
	St. Pierre	3	8	6	0.929 ± 0.084	0.0050 ± 0.0035
Italy	Pavia	3	9	7	0.944 ± 0.070	0.0065 ± 0.0043
	Brescia	3	9	9	1.000 ± 0.052	0.0134 ± 0.0081
	Castellanza	3	8	6	0.893 ± 0.111	0.0095 ± 0.0061
	Cesena	2	7	6	0.952 ± 0.096	0.0191 ± 0.0116
	Modena	2	5	4	0.900 ± 0.161	0.0080 ± 0.0058
Overall		26	76	52	0.986 ± 0.005	0.0134 ± 0.0072

Gene diversity: Equivalent to the expected heterozygosity for diploid data. It is defined as the probability that two randomly chosen haplotypes are different in the sample.

Nucleotide diversity: the probability that two randomly chosen homologous nucleotide sites are different.

An estimate of variability distribution in and among the nine tested population samples is reported in Table 3. AMOVA indicated that most of the variation occurs within individuals (74.36%) while only about 8% of total variation is detected among populations. The inter-individual variation within samples accounted for 17.51% (*P* < 0.05) of the total variance. When the three main geographical population groups (Thailand, Réunion and Italy) are accounted for (Table 4), similar and relatively low percentages of variation were observed among groups (8.12%) and among populations within groups (7.09%), indicating that the genetic similarity is not related to the geographic proximity of populations. Indeed, according to the SAMOVA analysis (Table 5) the best partitioning of genetic diversity was obtained when the nine samples were grouped into seven groups: *F*_CT_ =0.186, *P* = 0.011. Here the genetic diversity is not only among geographical regions, as even the two ancestral Thai populations are separated into two groups, while the North-Italian populations are separated into three groups and the two Réunion populations into two groups.

**Table 3:** **Hierarchical analysis of ITS2 molecular variance (AMOVA) among the 9** ***Aedes albopictus*** **samples (using Pairwise difference distance method)**

**Source of variation**	**d.f**	**Sum of squares**	**Variance components**	**Percentage of variation**	***P***
Among populations	8	125.064	0.64510 Va	8.13	0.0401
Among individuals within populations	17	166.936	1.38968 Vb	17.51	0.0020
Within individuals	50	295.000	5.90000 Vc	74.36	<0.0001
Total	75	587.000	7.93478

**Table 4:** **Hierarchical analysis of ITS2 molecular variance (AMOVA) comparing three geographical population groups: Thailand, Réunion, and Italy (using Pairwise difference distance method)**

**Source of variation**	**d.f.**	**Sum of squares**	**Variance components**	**Percentage of variation**	***P***
Among groups	2	55.591	0.66071 Va	8.12	0.0088
Among populations within groups	6	69.474	0.57664 Vb	7.09	0.0010
Within populations	67	461.936	6.89456 Vc	84.78	<0.0001
Total	75	587.000	8.13192

**Table 5:** **Spatial analysis of molecular variance (SAMOVA) for different population partitions**

**Number of groups (** ***K*** **)**	***F***_**CT**_	***P***	**Population partition**
2	0.1756	0.1124	(Ban Rai), (Phato, Pavia, Brescia, Castellanza, Cesena, Modena, St. Pierre, St. Denis)
3	0.1369	0.0274	(Ban Rai), (Phato, Brescia, Castellanza, Cesena, Modena, St. Pierre, St. Denis), (Pavia)
4	0.1406	0.0117	(Ban Rai), (Phato, Brescia, Castellanza, Cesena, Modena, St. Pierre), (St. Denis), (Pavia)
5	0.1471	0.0117	(Ban Rai), (Phato, Brescia, Castellanza, Cesena, Modena), (Pavia), (St. Pierre), (St. Denis)
6	0.1731	0.0010	(Ban Rai), (Phato, Cesena), (Brescia, Castellanza, Modena), (Pavia), (St. Pierre), (St. Denis)
7*	0.1861	0.0108	(Ban Rai), (Phato), (Brescia, Castellanza, Modena), (Cesena), (Pavia), (St. Pierre), (St. Denis)
8	0.1783	0.0655	(Ban Rai), (Phato), (Brescia, Modena), (Castellanza), (Cesena), (Pavia), (St. Pierre), (St. Denis)

*Number of groups (*K*) with the highest *F*_CT_ value.

### ITS2 haplotype phylogeny and population distribution

**Figure 1:**
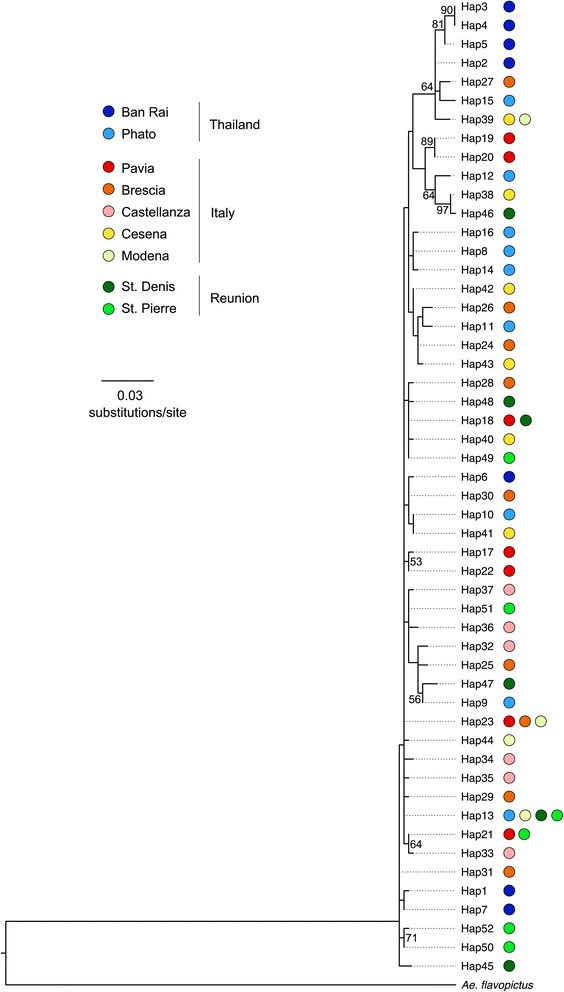
**Maximum Likelihood phylogeny of ITS2 haplotypes derived from the wild** ***Aedes albopictus*** **population samples with an** ***Ae. flavopictus*** **sequence [GenBank:AF353559] as outgroup.** The Kimura-2-parameter and Γ distribution model of molecular evolution was applied. Bootstrap values (1000 replicates) above 50% are indicated.

The phylogenetic analysis based on the 52 ITS2 haplotype sequences identified in the nine geographic populations resulted in a Maximum Likelihood tree with a log likelihood of −1428.3 (Figure 1). The ITS2 sequences form a compact cluster with very few nodes supported by bootstrap values greater than 50%. Highly related sequences are distributed across populations from different geographical regions; some evidence of clustering of tightly related sequences is present in Ban Rai, Phato (Thailand) and to a lesser degree in St. Pierre (Réunion).

The majority (47of 52) of the haplotypes are present in only one population. Only three haplotypes are shared by two populations (haplotype 18 in Pavia and St. Denis, haplotype 21 in Pavia and St. Pierre, and haplotype 39 in Cesena and Modena). Haplotype 23 is shared by three Italian populations (Pavia, Brescia and Modena), and haplotype 13 is present in four populations (Phato, Modena, St. Denis and St. Pierre).

### SSR: Isolation and characterization

Out of a total of 109 sequenced clones obtained from the *Ae. albopictus* library, 92 contained tandem DNA repeats with at least 5 repeat units. BLASTn analyses showed that one or both flanking regions were shared in 42 sequences and they were thus excluded from the subsequent characterization. Within the remaining 50 sequenced clones, five harboured more than one tandem DNA repeat, resulting in the identification of 55 microsatellite motifs. Among these, trinucleotide motifs were the most abundant (28), followed by dinucleotide (22), mononucleotide (3), heptanucleotide (1) and hendecanucleotide motifs (1). Among the trinucleotides motifs, CAA was highly abundant (17) followed by TGA (10). Among the dinucleotide motifs, TG was predominant (14) followed by CT (8). Thirty-seven of these microsatellite motifs contained perfect repeat arrays. Twelve microsatellites contained imperfect repeats and the other six microsatellites contained compound repeats.

Forty-five microsatellite sequences were considered for further validation, as the others lacked sufficient flanking regions to design primers. After the PCR screening on 20 individuals from the Rimini laboratory strain, 23 SSR sequences were validated as loci (Table 6). The nucleotide sequences of these validated SSR loci were submitted to GenBank [Genbank: KP859591-KP859613].

**Table 6:** **The 23** ***Aedes albopictus*** **microsatellite loci validated in this study**

**Locus**	**Type** ^**1**^	**Repeat motif**	**Primer sequences (5’ to 3’)** ^**2**^	***T***_**a**_ **(°C)** ^**3**^
**Aealbmic1**	P	(TC)_10_	F: [6FAM]ACCTTGCGTAGGATGACGAT	60
			R: GCCATGATCACGAGCCTATT	
**Aealbmic2**	I	(GTT)_5_TGGAGTGAG(GTT)_6_	F: [6FAM]ACGATGCGTAACCATTCGAT	58
			R: AACACCGCCGAATATGAAAC	
**Aealbmic3**	P	(AAC)_9_	F: [HEX]ACCATACAGCCTGGAGTTCG	58
			R: GGGGTTGTGTGAATTGTCGT	
**Aealbmic4**	P	(CAA)_8_	F: [6FAM]ATCGCGGGTTTTCTATTCCT	58
			R: ATCAACGAAACCGAAAGCAT	
**Aealbmic5**	P	(TGT)_13_	F: [HEX]AACCCATCGAACACAGAAGG	58
			R: GTACGGTTGACTCGCTGTGA	
**Aealbmic6**	I	(GTT)_3_GCT(GTT)_3_ GGT(GTT)_4_	F: [HEX]GATGGTCCGTATTTGGGTTG	57
			R: ATCTTCACTCATCCGCCATC	
**Aealbmic7**	I	(TTG)_8_ATG(TTG)_4_	F: [HEX]ATAGACGGGAGTCGGTTCCT	58
			R: TCCAACCGCTAGTGTCATCA	
**Aealbmic8**	P	(GT)_8_	F: [HEX]TTGTTGTTCGGTTGTTGTTTG	57
			R: CGGGTTCCAACTATGTACGA	
**Aealbmic9**	P	(GAT)_7_	F: [HEX]GCGATGACAGTGGAACAAGA	58
			R: GCTTGGCAGGGAACAAATTA	
**Aealbmic10**	P	(ATC)_9_	F: [6FAM]ATCGCCTTCACTCTTCTTCG	60
			R: CCAATCCTGAGCCGTACATT	
**Aealbmic11**	P	(TGT)_5_	F: [6FAM]CTCTGCGTTCCGGTTCTATC	58
			R: AGGCAACCTCTCGAATGAAA	
**Aealbmic12**	P	(GAT)_7_	F: [6FAM]AGAGCCCTCGAAAAGAGAGC	58
			R: AGCACTCATTCTTGGCTTGG	
**Aealbmic13**	I	(GAT)GAC(GAT)_4_	F: [6FAM]TCACACCATGGTCAAAGCAT	58
			R: TGCTGAGTTGAATGGAAACG	
**Aealbmic14**	I	(CT)_11_ T(CT)_2_	F: [6FAM]CAGAAGGTCTTGGATTCACTC	58
			R: TTTCCAATTCGTTTCTGGTTC	
**Aealbmic15**	P	(GTT)_7_	F: [6FAM]GGAATGGTTCCCTGGCTAAT	58
			R: CCAACTCCGAAGAAGCCATA	
**Aealbmic16**	P	(CAT)_7_	F: [HEX]CACAACAACGAGAGTGTCGAA	60
			R: CCGAGGGCAACACGATATAC	
**Aealbmic17**	P	(CAA)_15_	F: TCACCACAACAAACGGAATC	58
			R: AGAGTTTTGGGCGCAGTTTA	
**Aealbmic18** ^**4**^	I	(TG)_12_TA(TG)_7_	F: GGGCCGAATCTCATATACCA	59
			R: CTCAAATCGGTTGACAGACG	
**Aealbmic19**	P	(ATC)_9_	F: ACATATAGGTAATCCTTGCGCTGAT	59
			R: GTTCCCGTATTTGATATTTGCTTT	
**Aealbmic20**	P	(CA)_44_	F: GGAGGCCATCATAATTTCGAC	58
			R: AGTGGACCACAGACAGACCATT	
**Aealbmic21**	P	(ATG)_7_	F: CCCTACAGCCCTGATTGAGA	58
			R: CGAGTTGGGATGTGTGATTG	
**Aealbmic22** ^**5**^	I	(AC)_45_AT(AC)_2_AT(AC)_5_	F: AAATCGGTTGAGGTTTACTGC	59
			R: ATTCTTAGCCGGCAACTACG	
**Aealbmic23**	P	(ACA)_8_	F: AACGGAGCGGAGTCGATTAT	58
			R: CTACTACCCGCTGCCTTCTG	

^1^Motif type (P: perfect repeats; I: imperfect repeats).

^2^The fluorescent label (6FAM or HEX) on the forward primer is indicated for the 16 loci that were subsequently used for the population analysis.

^3^Annealing temperature (*T*_a_).

^4,5^SSR sequences that correspond to previously identified loci: (alb212) [57] and (AealbD2) [58], respectively.

### Evaluation of microsatellite polymorphism in ancestral and adventive populations

On the basis of a preliminary screening performed on the Rimini strain, 16 (Aealbmic1 to Aealbmic16) of the 23 validated SSR loci (Table 6) were used to analyse the degree of polymorphism in 79 mosquitoes from three populations: Ban Rai (Thailand), Brescia and Cesena (Italy) (Table 7). The average Polymorphic Information Content (PIC) estimated across the 16 loci was 0.56 ± 0.29 in Ban Rai, 0.50 ± 0.26 in Cesena and 0.47 ± 0.21 in Brescia, suggesting that these loci are informative for population analyses. Heterozygous individuals were present in both sexes for all 16 loci, excluding any condition of sex linkage. Tests for Hardy-Weinberg equilibrium (HWE), after sequential Bonferroni correction [58], revealed that five loci did not meet the expectations of this model (Table 7): three (Aealbmic4, 11, 13) in the Ban Rai sample, one in Cesena (Aealbmic3) and two in Brescia (Aealbmic13, 16). However, the locus/population combinations that were not in HWE were not concentrated at any locus or in any population. As no evidence of linkage disequilibrium between loci was assessed, these 16 loci can be considered to be independent. In the three populations most of the loci display relatively high levels of gene diversity (Hs). The frequency of null alleles across the loci was generally relatively low apart from Aealbmic15 with an estimate of 0.98 in Cesena. Two alleles were detected in Cesena at the Aealbmic15 locus, but one was very rare. In the other populations this locus was monomorphic for the common allele. Aealbmic1 is monomorphic in Ban Rai and Cesena, while in Brescia two alleles were present. Across the loci a higher level of polymorphism was observed in Ban Rai, with a mean number of alleles per locus of 6.19 (Table 7). Private alleles (n_p_) were detected in all populations, with the highest average value in the Ban Rai sample (2.25). Analysis of molecular variance (AMOVA) across the three populations indicates that the greatest portion of variance, 76.62%, is found within individuals, while 16.80% is among individuals and only the remaining 6.57% is among populations. The F_ST_ values between each pair of populations are statistically significant and indicate that the degree of differentiation between the two Northern Italian samples, Cesena and Brescia (F_ST_ = 0.061), is not very different from those between these two Italian samples and the Ban Rai sample (F_ST_ = 0.072 in both comparisons).

**Table 7:** **Microsatellite variation in three** ***Aedes albopictus*** **populations**

**Locus**	**Ban Rai (n = 24)**									**Cesena (n = 31)**									**Brescia (n = 24)**								
	**n**_**a**_	**n**_**e**_	**n**_**p**_	**H**_**O**_	**H**_**E**_	**Hs**	**F**_**IS**_	**PIC**	**A**_**n**_	**n**_**a**_	**n**_**e**_	**n**_**p**_	**H**_**O**_	**H**_**E**_	**Hs**	**F**_**IS**_	**PIC**	**A**_**n**_	**n**_**a**_	**n**_**e**_	**n**_**p**_	**H**_**O**_	**H**_**E**_	**Hs**	**F**_**IS**_	**PIC**	**A**_**n**_
**Aealbmic1**	1	1.00	0	0.00	0.00	0.00	-	0.00	-	1	1.00	0	0.00	0.00	0.00	-	0.00	-	2	1.28	1	0.25	0.22	0.22	−0.14	0.20	0.00
**Aealbmic2**	4	2.44	1	0.50	0.59	0.60	0.15	0.50	0.03	4	1.90	1	0.45	0.47	0.48	0.04	0.40	0.03	2	1.99	0	0.58	0.50	0.51	−0.16	0.37	0.00
**Aealbmic3**	13	8.35	7	0.75	0.88	0.90	0.15	0.87	0.09	8	4.49	1	0.45	0.78*	0.80	0.42	0.76	0.21	4	2.53	0	0.70	0.60	0.62	−0.17	0.55	0.00
**Aealbmic4**	6	3.54	2	0.42	0.72*	0.74	0.42	0.68	0.30	5	4.21	0	0.71	0.76	0.78	0.07	0.72	0.19	4	2.81	0	0.58	0.64	0.66	0.09	0.59	0.18
**Aealbmic5**	6	3.89	3	0.50	0.74	0.76	0.32	0.70	0.14	8	2.27	2	0.39	0.56	0.57	0.30	0.54	0.13	6	3.59	3	0.44	0.72	0.74	0.39	0.67	0.22
**Aealbmic6**	7	2.16	1	0.55	0.54	0.55	−0.02	0.51	0.00	4	2.75	0	0.52	0.64	0.65	0.19	0.58	0.12	4	2.97	0	0.58	0.66	0.68	0.12	0.60	0.16
**Aealbmic7**	6	3.87	2	0.79	0.74	0.76	−0.07	0.70	0.00	5	2.44	2	0.73	0.59	0.60	−0.24	0.53	0.00	4	2.77	2	0.63	0.64	0.65	0.02	0.57	0.00
**Aealbmic8**	8	5.10	2	0.83	0.80	0.82	−0.04	0.78	0.02	7	3.94	1	0.87	0.75	0.76	−0.16	0.71	0.00	5	3.89	0	0.79	0.74	0.76	−0.07	0.70	0.03
**Aealbmic9**	10	4.52	6	0.67	0.78	0.80	0.14	0.76	0.10	8	4.73	0	0.81	0.79	0.80	−0.03	0.76	0.11	3	2.11	0	0.54	0.53	0.54	−0.02	0.42	0.00
**Aealbmic10**	4	2.85	1	0.50	0.65	0.67	0.23	0.58	0.11	4	2.26	1	0.70	0.56	0.56	−0.25	0.46	0.00	3	1.97	0	0.44	0.49	0.51	0.10	0.39	0.04
**Aealbmic11**	9	4.18	4	0.39	0.76*	0.79	0.49	0.73	0.23	6	3.11	0	0.58	0.68	0.69	0.15	0.63	0.06	4	2.58	0	0.41	0.61	0.63	0.33	0.54	0.15
**Aealbmic12**	7	4.65	2	0.55	0.79	0.81	0.30	0.75	0.18	6	4.51	0	0.61	0.78	0.79	0.22	0.74	0.09	6	4.23	0	0.65	0.76	0.78	0.14	0.73	0.07
**Aealbmic13**	8	3.95	3	0.46	0.75*	0.77	0.39	0.72	0.17	8	2.74	2	0.55	0.63	0.65	0.13	0.60	0.10	4	2.31	0	0.21	0.57*	0.59	0.63	0.52	0.24
**Aealbmic14**	2	1.04	1	0.04	0.04	0.04	0.00	0.04	0.00	1	1.00	0	0.00	0.00	0.00	-	0.00	-	3	1.20	2	0.09	0.17	0.17	0.47	0.16	0.20
**Aealbmic15**	1	1.00	0	0.00	0.00	0.00	-	0.00	-	2	1.07	1	0.00	0.06	0.07	1.00	0.06	0.98	1	1.00	0	0.00	0.00	0.00	-	0.00	-
**Aealbmic16**	7	3.60	1	0.70	0.72	0.74	0.03	0.68	0.00	6	2.17	2	0.61	0.54	0.55	−0.13	0.49	0.00	5	2.54	1	0.33	0.61*	0.63	0.46	0.56	0.16
**Mean**	6.19	3.51	2.25	0.48	0.59	0.61	0.18	0.56		5.19	2.79	0.81	0.50	0.54	0.55	0.12	0.50		3.75	2.49	0.56	0.45	0.53	0.54	0.15	0.47	
**SD**	3.25	1.85	1.98	0.27	0.30	0.31		0.29		2.40	1.27	0.83	0.28	0.27	0.28		0.26		1.39	0.92	0.96	0.22	0.22	0.22		0.21	

Number of individuals (n), number of alleles (n_a_), number of effective alleles (n_e_), number of private alleles (n_p_), observed (H_O_), expected (H_E_) heterozygosities, gene diversity (H_S_), inbreeding index (F_IS_), Polymorphic Information Content (PIC) and null allele frequencies (A_n_) per locus are reported. The asterisks indicate significant departures from Hardy-Weinberg equilibrium after Bonferroni correction at *P* < 0.05.

## Discussion

In the absence of a fully annotated genome, molecular markers for *Ae. albopictus* are crucially needed to track the rapid spread of this mosquito that is causing increasing concern due to its status as a vector of several arboviruses of public health importance [3,9]. The two types of genetic markers here implemented give a picture of the distribution of genetic diversity within and between populations providing clues on the dispersion dynamics of this species. ITS2 sequence variation has been analysed in mosquitoes from the supposed native range, Thailand, from an old colonised area, Réunion, and from a newly invaded area, Italy. It appears that substantial variability, both in terms of haplotype number and gene diversity, is present in the native as well in the two areas of introduction. The haplotype variability is mostly concentrated within individuals (74.36%) or within populations (84.78%), while only a relatively small proportion into geographical differences, both at single population (8.13%) and at geographic area level (8.12%). This implies that, according to SAMOVA, the haplotype variation, besides being high, has a distribution that is independent from geography. Different haplotypes are present in each individual of the nine samples, and the tree clearly demonstrates that they are similar and dispersed across native and adventive populations (Figure 1). Substantially concordant conclusions can be obtained with the newly developed SSR markers, which display a high level of polymorphism both in the ancestral (Ban Rai, Thailand) and adventive populations (North Italian samples), previously scored for ITS2 variability. Although the chromosomal location of these markers remains unknown, the assessed linkage equilibrium between them suggests that they are statistically independent and their variability patterns might reflect genome wide patterns across populations. Based on the population samples here analysed, the SSR markers were, as expected, more informative than ITS2 in revealing the slight genetic diversity between native and derived populations both in terms of variability and differentiation. About 7% of total variability (AMOVA) is represented by the differences between the three populations, while the highest variability was found within populations. This is an agreement with a previous study on mosquitoes collected in Réunion [59]. The F_ST_ estimates indicate that the differentiation between the two Italian samples is slightly smaller (F_ST_ = 0.061) than that between the Italian and Thai samples (F_ST_ = 0.072), suggesting the absence of great differentiation between ancestral and derived populations. The ancestral nature of the Thai population is also corroborated by its high level of variability.

Taken together these genetic data raise questions on the way this mosquito is spreading and becoming established worldwide. Clearly, our data indicate that the diffusion of this mosquito does not conform to a progressive expansion away from the native Asian source area, but supports a relatively recent and chaotic propagule distribution mediated by human activities. Under this scenario, multiple introductions and admixture events probably play an important role in maintaining the genetic diversity and in avoiding bottleneck effects.

## Conclusions

In this study we have exploited nuclear molecular markers that have allowed us to highlight the presence of high intraspecific variability and a low/moderate level of differentiation of *Ae. albopictus* populations collected in different eco-geographic areas. Most of the genetic variation was detected at the individual level and this contributed to the high genetic variation observed within populations. This genetic feature, also revealed using other markers [7,34,36,59] may have facilitated the characteristic ability of this invasive species to expand and adapt to novel environments.

The highly polymorphic SSR markers here implemented, together with the set previously described [36,59,60], represent an important tool for reconstructing the routes of invasion and for the identification of the origins of mosquito outbreaks.
